# Protocol for immunofluorescence staining and large-scale analysis to quantify microglial cell morphology at single-cell resolution in mice

**DOI:** 10.1016/j.xpro.2024.103467

**Published:** 2024-12-04

**Authors:** Frida Lind-Holm Mogensen, Corrado Ameli, Alexander Skupin, Alessandro Michelucci

**Affiliations:** 1 Neuro-Immunology Group, Department of Cancer Research, Luxembourg Institute of Health, 6A, Rue Nicolas-Ernest Barblé, L-1210 Luxembourg, Luxembourg; 2 Faculty of Science, Technology and Medicine, University of Luxembourg, 2, avenue de l’Université, L-4365 Esch-sur-Alzette, Luxembourg; 3 Integrative Cell Signalling Group, Luxembourg Centre for Systems Biomedicine, University of Luxembourg, 7, Avenue des Hauts-Fourneaux, L-4362 Esch-sur-Alzette, Luxembourg; 4 Department of Neurosciences, University of California, San Diego, 9500 Gillman Drive, La Jolla, CA 92093, USA; 5 Integrative Biophysics, Department of Physics and Material Science, University of Luxembourg, 162a, Avenue de la Faïencerie, L-1511 Luxembourg, Luxembourg

**Keywords:** bioinformatics, cell biology, immunology, neuroscience

## Abstract

Here, we present a protocol for quantifying microglial cell morphology at the single-cell level in mice. We provide comprehensive details, starting from optimal mouse brain dissection to computational analyses of up to 350 microglial cells per brain slice. Analyzing the morphology of microglial cells is essential for understanding their functional and activation states in different conditions, including during disease development and progression, as well as for assessing the effect of therapeutic interventions.

For complete details on the use and execution of this protocol, please refer to Lind-Holm Mogensen et al.^1^ and Fixemer et al.^2^

## Highlights

• Protocol to quantify mouse microglial cell morphology at single-cell level *in situ*
• Combines precise tissue handling, staining, and semi-automated imaging analyses
• Allows extraction of 16 morphological features from up to 350 cells per image
• Enhances understanding of microglial cell phenotypic acquisition and dynamics

## Abstract (editor highlights)

Publisher’s note: Undertaking any experimental protocol requires adherence to local institutional guidelines for laboratory safety and ethics.

## Graphical abstract

**Figure:**
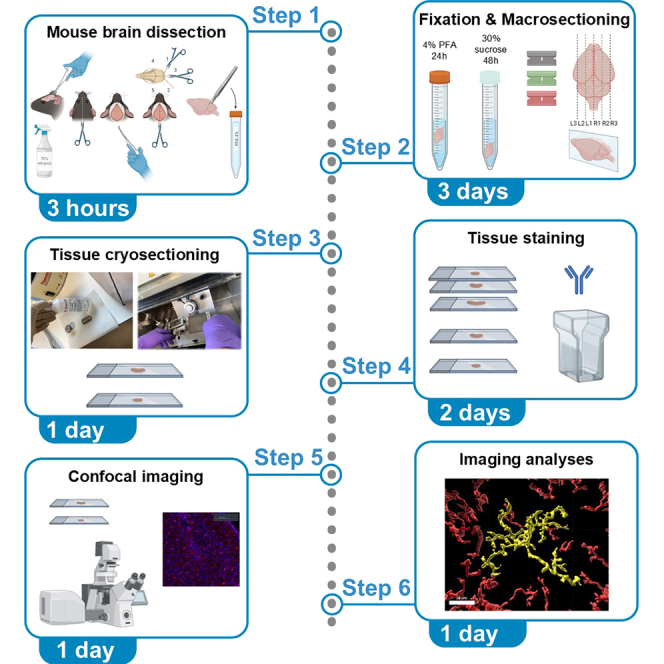

## Before you begin

In fundamental and preclinical research, characterizing new animal models of human diseases and evaluating the efficacy of emerging drugs requires meticulous assessment techniques. Microglia morphology serves as an important metric in these efforts, serving not only as a marker for disease progression, but also as a key indicator of treatment response in the brain. This protocol outlines a detailed methodology aimed at quantifying microglial cell morphology at the single-cell level *in situ*. Through a combination of precise tissue handling, staining techniques and semi-automated imaging analyses, this protocol offers a comprehensive way to assess microglial morphological features, including number of processes, volume, and length of processes and clustering of various phenotypic shapes, such as compactness (e.g., amoeboid features). We previously used the fully automated pipeline in mouse and human post-mortem tissues.^2^^,^^3^ We recently adopted an optimized protocol to characterize the role of microglia in a DJ-1 deficient mouse model and to specifically assess the effect of lipopolysaccharide (LPS)-induced neuroinflammation in wild-type and DJ-1 knockout mice.^1^ This protocol adds solutions to troubleshooting steps for tissue handling, staining and analysis, helping in optimizing microglia analyses *in situ* in murine brain samples at a single cell level. Additionally, the usage of one hemisphere for morphological analyses allows conducting simultaneous -omics studies on the other hemisphere, including single-cell transcriptional analyses. By integrating these methodologies, researchers can obtain valuable insights into microglial phenotypic acquisition and dynamics using a limited number of animals. Furthermore, this protocol offers the option to cluster cells based on morphology and in parallel conduct complementary analyses of microglial cells or other CNS features from the same brain. Taken together, incorporating high-resolution and high-throughput morphological analyses of microglial cells at the single cell level provides an important layer of information that enhances the understanding of microglia’s role in the palette of their phenotypic characterization in the conditions being studied.

### Institutional permissions

This protocol requires tissue sections from mouse models. All mouse experiments must be conducted under ethical approvals by the internal animal welfare structures and the national authorities responsible for animal experimentation before starting these procedures. Animal experiments described in this protocol were approved following the European Directive 2010/63/EU and Grand-Ducal Regulation of 11^th^ of January 2013 under the approval of animal license (LUPA2022/1) from the Animal Experimentation Ethics Committee at the University of Luxembourg (AEEC) and the Ministry of Agriculture, Viniculture and Rural Development.

## Key resources table

**Table:** 

REAGENT or RESOURCE	SOURCE	IDENTIFIER
**Antibodies**		

Rabbit anti-ionized calcium binding adaptor molecule 1 (IBA1) 1:1,000	Wako/Fujifilm	Cat#019–19741; RRID:AB_839504
Chicken anti-tyrosine hydroxylase (TH) 1:1,000	Abcam	Cat#ab76442; RRID:AB_1524535
Alexa Fluor 488 1:1,000	Thermo Fisher Scientific	Cat#A-11039; RRID:AB_2534096
Alexa Fluor 647 1:1,000	Thermo Fisher Scientific	Cat#A-21244; RRID:AB_2535812

**Chemicals, peptides, and recombinant proteins**		

PFA 4%	Alfa Aesar	Cat#J61899; CAS: 30525-89-4
PBS without Ca^2+^ Mg^2+^ 10x	RotiCell, Roth or of user choice	Cat#9150.1
0.9% Sodium chloride injection solution	Braun	Cat#L8002
Triton X-100	Thermo Fisher Scientific or of user choice	Cat#A16046.AE; CAS: 9002-93-1
Hydrogen peroxide 30% (H_2_O_2_ 30%)	Sigma-Aldrich or of user choice	Cat#216763; CAS: 7722-84-1
Bovine serum albumin	Sigma	Cat#A9418; CAS: 9048-46-8
Sucrose	Sigma or of user choice	Cat#SO389; CAS: 57-50-1
Fluoromount with DAPI	Invitrogen	Cat#00–4959-52
Lipopolysaccharide *E. coli* O55:B5	Sigma	Cat#L6529-1MG
Transparent nail polish	Maybelline or other brand of choice	N/A

**Experimental models: Organisms/strains**		

Mouse: C57Bl/6NCrl (SPF facility) 4 months old males	Charles River	RRID: IMSR_CRL:027

**Software and algorithms**		

Black Zen	Zeiss	2.3 SP1; RRID:SCR_018163
ZEISS ZEN Microscopy Software	Zeiss	RRID:SCR_013672
Imaris	Oxford Instruments	RRID:SCR_007370
Excel	Microsoft	N/A
Prism version 10.0.03	N/A	RRID:SCR_005375
MATLAB	MathWorks	RRID:SCR_001622
Imaris file converter	Oxford Instruments	RRID:SCR_007370
Imaris Stitcher 9 onward	Oxford Instruments	RRID:SCR_007370

**Other**		

LSM880 Confocal microscope	Zeiss	RRID:SCR_020925
Cryostat	Leica	Leica CM 1850 UV cryostat, RRID:SCR_025401
Rodent brain matrix	ASP Instruments, Kent Scientific	RBM-2000C
Kimwipes	Kimtech Science	Ref. 7216
OCT	Tissue-Tek	4583; CAS: 25322-68-3
Epredia Superfrost Plus Adhesion	Epredia	J1800AMNZ
Biopsy Zip-Seal bags (60 × 80 mm)	VWR	Cat#129-2001
Glass coplin box	Of user choice	N/A
Parafilm 100 mm	Parafilm	CNP8.1
Oil for microscopy, Zeiss Immersol 518 F	Fisher Scientific (Zeiss)	10539438; CAS : 195371-10-9
Cover slides 24 × 55 mm	Corning	2975–245
Scissors and forceps	Of user choice	N/A
Coverslips 12 mm, 0.13–0.16 mm	Epredia	CB00120RA120MNZ0

## Materials and equipment

### Embedding solution

• Prepare sucrose embedding solution by adding 30 g of sucrose on a weighing boat to ensure the correct amount.
• Subsequently add it to 50 mL of Milli-Q water.
• Mix with a magnetic stirrer and add up to 100 mL of Milli-Q water.

**Table:** 

Reagent	Final concentration	Amount
Sucrose	30%	30 g
Milli-Q water	N/A	To 100 mL
**Total**	**N/A**	**100 mL**

Store at +4°C for one month.

### Permeabilization and peroxidase removal buffer

• Prepare permeabilization and peroxidase removal buffer by adding 10 mL of H_2_O_2_ 30% to 88.5 mL of 1x PBS.
• Add 1.5 mL of Triton-X-100.
• Mix with a magnetic stirrer until fully dissolved.

**Table:** 

Reagent	Final concentration	Amount
H_2_O_2_ 30%	3%	10 mL
Triton-X-100	1,5%	1.5 mL
PBS 1x	N/A	88.5 mL
**Total**	**N/A**	**100 mL**

Prepare daily (H_2_O_2_ is not stable).

**CRITICAL:** H_2_O_2_ is corrosive, oxidizing and irritant. Wear appropriate individual protective equipment. H_2_O_2_ should not be released into the environment.

**CRITICAL:** Triton-X-100 is viscous. Pipette it slowly with the “reverse pipetting method”. This means you have to push the pipette plunger up to the second stop, then aspirate the liquid and push the plunger to the first stop to release the liquid. Do not dispense the remaining liquid in the tip. It can be trashed with the tip or put back in the original source.

***Alternatives:*** Triton-X-100 can be substituted by Tween 20.

**Table:** Wash buffer 1

Reagent	Final concentration	Amount
Triton-X-100	0,1%	0.1 mL
1x PBS	N/A	99,9 mL
**Total**	**N/A**	**100 mL**

Store at room temperature.

**CRITICAL:** Triton-X-100 is viscous. Pipette it slowly with the “reverse pipetting method”. This means you have to push the pipette plunger up to the second stop, then aspirate the liquid and push the plunger to the first stop to release the liquid. Do not dispense the remaining liquid in the tip. It can be trashed with the tip or put back in the original source.

***Alternatives:*** Triton-X-100 can be substituted by Tween 20.

### Blocking buffer

• Prepare blocking buffer by adding 5 g of BSA powder to 100 mL of 1× PBS.

**Table:** 

Reagent	Final concentration	Amount
Bovine serum albumin (BSA)	5%	5 g
1x PBS	N/A	To 100 mL
**Total**	**N/A**	**100 mL**

• Add BSA on the day of the experiments and store for maximum two weeks at +4°C after adding BSA.

***Note:*** BSA powder may take time to dissolve. Prepare the solution one hour before use. Avoid foaming: mix gently by stirring or with a rolling shaker.

### Antibody dilution buffer

• Prepare antibody dilution buffer by adding 0.3 mL of Triton-X-100 and 2 g of BSA powder to 100 mL of 1× PBS.

**Table:** 

Reagent	Final concentration	Amount
Triton-X-100	0.3%	0.3 mL
BSA	2%	2 g
1x PBS	N/A	To 100 mL
**Total**	**N/A**	**100 mL**

Pre-buffer can be prepared without BSA and stored at room temperature. Add BSA on the day of the experiment and store for maximum two weeks at +4°C after adding BSA.

***Alternatives:*** Triton-X-100 can be substituted by Tween 20.

**CRITICAL:** Triton-X-100 is viscous. Pipette it slowly with the “reverse pipetting method”. This means you have to push the pipette plunger up to the second stop, then aspirate the liquid and push the plunger to the first stop to release the liquid. Do not dispense the remaining liquid in the tip. It can be trashed with the tip or put back in the original source. We recommend to prepare 500 mL of 0.3% Triton-X-100 in PBS as a stock and then, on the day of the staining, take out the desired volume for the staining and add BSA to that.

***Note:*** BSA powder may take time to dissolve. Prepare the solution one hour before use. Avoid foaming: mix gently by stirring or with a rolling shaker.

**Table:** Wash buffer 2

Reagent	Final concentration	Amount
Triton-X-100	0,3%	0.3 mL
1x PBS	N/A	99,7 mL
**Total**	**N/A**	**100 mL**

Store at room temperature.

***Alternatives:*** Triton-X-100 can be substituted by Tween 20.

**CRITICAL:** Triton-X-100 is viscous. Pipette it slowly with the “reverse pipetting method”. This means you have to push the pipette plunger up to the second stop, then aspirate the liquid and push the plunger to the first stop to release the liquid. Do not dispense the remaining liquid in the tip. It can be trashed with the tip or put back in the original source.

## Step-by-step method details

### Animal and tissue preparation

**Timing: 3 h**

1. Inspect the mice and weigh each one to calculate injection dose.
2. Intraperitoneally inject ketamine (100 mg/mL, Nimatek Vet) and medetomidine (1 mg/mL, Dorbene Vet) diluted in saline solution.
  a. Cover the animal with paper to dim the light and keep it in its well-known cage.
  b. Meanwhile prepare a clean dissection setup by cleaning the designated dissection table with Bacillol followed by removal and cleaning with 70% ethanol.
  c. Prepare three pieces of tissue paper.

***Note:*** The first tissue paper will be added on top of the drip tray, the second will be placed next to the drip tray and syringe, autoclaved forceps, bone trimmer and scissors will be placed on top. The third piece will be used to cover the mouse and to clean it.

**Figure:**
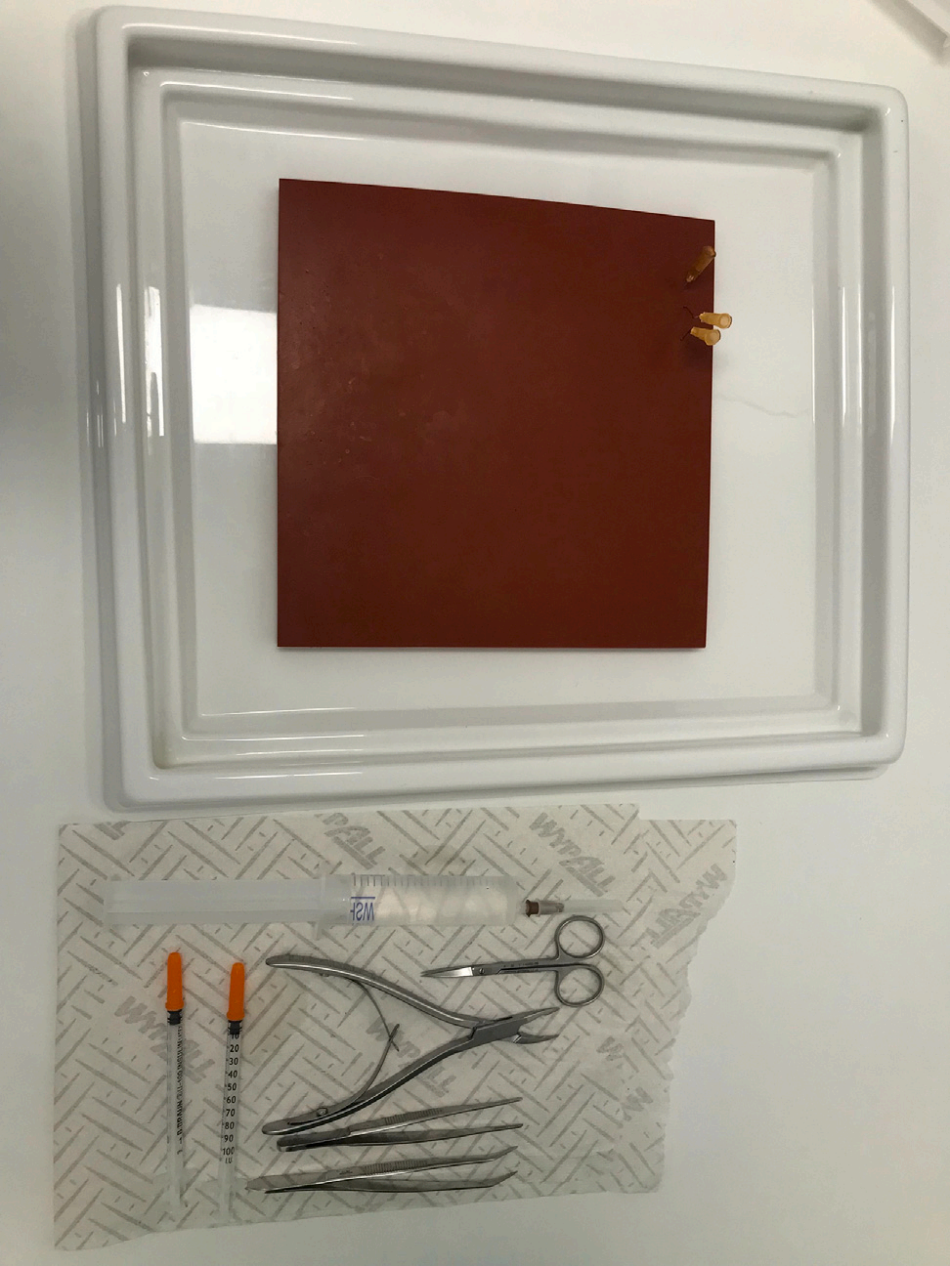

.

3. Assess reflexes and clean the animal with ethanol. When no reflexes are observed, open up the chest, break the diaphragm and do a cardiac perfusion with cold PBS.
4. Dissect out the brain using forceps, bone trimmer and scissors following the steps below.

**Figure:**
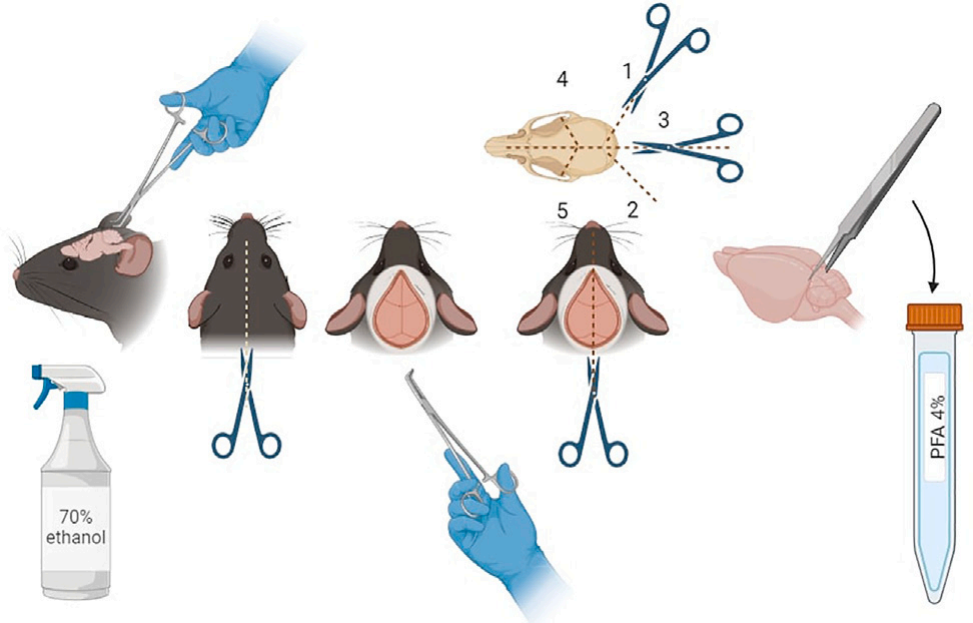

  a. Cut along the intraparietal bone on your right (1) and then on your left (2).
  b. Subsequently make a 5 mm cut in the middle and with a bone trimmer or forceps remove the occipital and intraparietal bone.
  c. Then with the blunt part of the scissor facing the brain tissue, cut along the midline all along to the frontal bone and to the nasal bone if possible (3). Then cut on your right between the parietal bone and frontal bone (4) to the eye sockets to loosen the bone. Do the same on your left (5).
  d. Remove as much bone as possible without touching any brain tissue. By turning the head upside down, the brain should now be able to be loosened out gently. Use forceps or tweezers very gently and place the brain into a 15 mL Falcon tube.

***Note:*** If desired, one hemisphere can be used for histology for microglial morphology analysis and the other for RNA or protein extraction to allow for side-by-side comparison between microglial images and gene expression. If the whole brain will be used for histology, we suggest to perfuse directly with cold 4% PFA.

5. If desired, the brain can be washed in a Petri dish with cold PBS and immediately after cut into two hemispheres using a rodent brain matrix.
  a. Drop-fix one hemisphere in cold 4% PFA and keep it at 4°C for 24 h.
  b. Put the other hemisphere in PBS for further downstream analyses.
6. Immediately store on ice.

**Pause Point:** [If you would like to use the whole brain for extraction of morphological features of microglia, you can now pause for 48 hours while the brain is undergoing fixation. If you would like to use one hemisphere for further analyses, you can now directly process the hemisphere of interest and leave the other hemisphere in the fridge for 24 hours.]

### Use of one hemisphere for further analyses

Depending on the scientific question, you can use just one hemisphere to perform the microglial morphological feature extraction. Thus, if you would like to carry out further analyses or experiments with the other hemisphere, it will be important to separate the two hemispheres using the rodent brain matrix.

If desired, you can for example extract RNA or proteins if you wish to compare protein or RNA expression with microglial morphological features from the same brain.

### Fixation and embedding

**Timing: 24–48 h of fixation; 48 h for sucrose embedding; 1 h preparation time**

7. Keep the brain hemisphere in 4% PFA at 4°C for 24–36 h.
8. Discard the 4% PFA solution in an appropriate container under a chemical hood.
  a. Wash gently 3x with cold PBS to remove all PFA.
  b. Pre-embed the tissue in 30% sucrose in Milli-Q water at 4°C.

***Note:*** Embedding in sucrose lasts until the brain is at the complete bottom of the 15 mL Falcon tube, which for a mouse brain usually takes 48 hours. It is crucial to embed the brain tissue completely in sucrose before cutting.

9. Remove all 30% sucrose solution without drying the brain completely. Dry gently the brain with Kimwipes and place it in the rodent brain matrix.
10. Place 5 sterile blades ready to be inserted in the brain matrix.
  **Figure:**
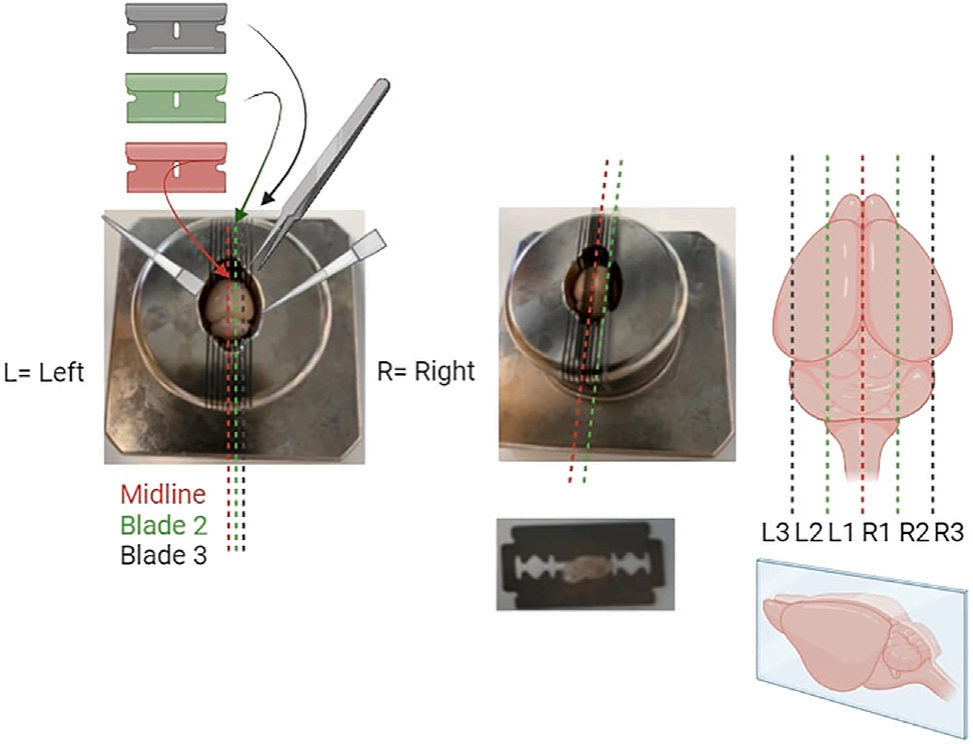

  a. Use forceps to keep the brain completely aligned in the matrix and/or use the large part of a sterile pipet tip to place the brain completely in the middle.
  b. When it is completely aligned, add the five blades to the brain matrix.
  c. To make sure the brain is cut via the midline; push down the middle blade 2 mm to fix the brain.
  d. Push down all the blades in one movement to cut the brain into macro-sections starting from the mid line.
    ***Note:*** If desired, it is possible to cut the brain into ten macro-sections via nine blade insertions in the brain matrix. However, it will be a challenge to cut smaller micro-sections on the cryostat. We recommend the use of the mouse brain atlas^4^ to identify brain regions of interest before cutting.
11. Add the brain or macro-sections to a labeled biopsy bag with ziplock on ice and store at − 80°C until further usage.
12. To make sure that the brain is completely frozen before cutting, keep at − 80°C for at least 48 h.

**CRITICAL:** To avoid fixing in paraffin, you can substitute with sucrose solution, which will make crosslinks and make the tissue harder and easier to cut. It is important to only change the tissue from 4°C to − 80°C when completely embedded in sucrose.

**Pause Point:** [To ensure that the brain is completely frozen, we recommend keeping it at − 80°C for 2 days, but longer storage is also possible until further use.]

### Tissue sectioning using a cryostat

**Timing: 48 h freezing prior to cutting; 1–3 h cutting**

This step is needed to cut macro sections into 50 μm tissue sections and accurately label each slide with the slide number with approximate coordinates and mouse ID, followed by storing them for future use.

13. Turn on the cryostat and make sure it is set at − 20°C.
14. While waiting for the cryostat to cool down, label well the slides for collection of tissue with a pencil or use sticker labels.

***Note:*** We suggest the following naming: MouseID_Genotype_Sex_Hemisphere_Number of macrosection_Number of section within that macrosection. Example: DO007_WT_M_L_3/18. Use the Mouse Brain Atlas^4^ to orient from start to end where to cut.

15. Embed the desired macro-section in OCT (Tissue-Tek) on its designated holder. Make sure there are no bubbles.

**Figure:**
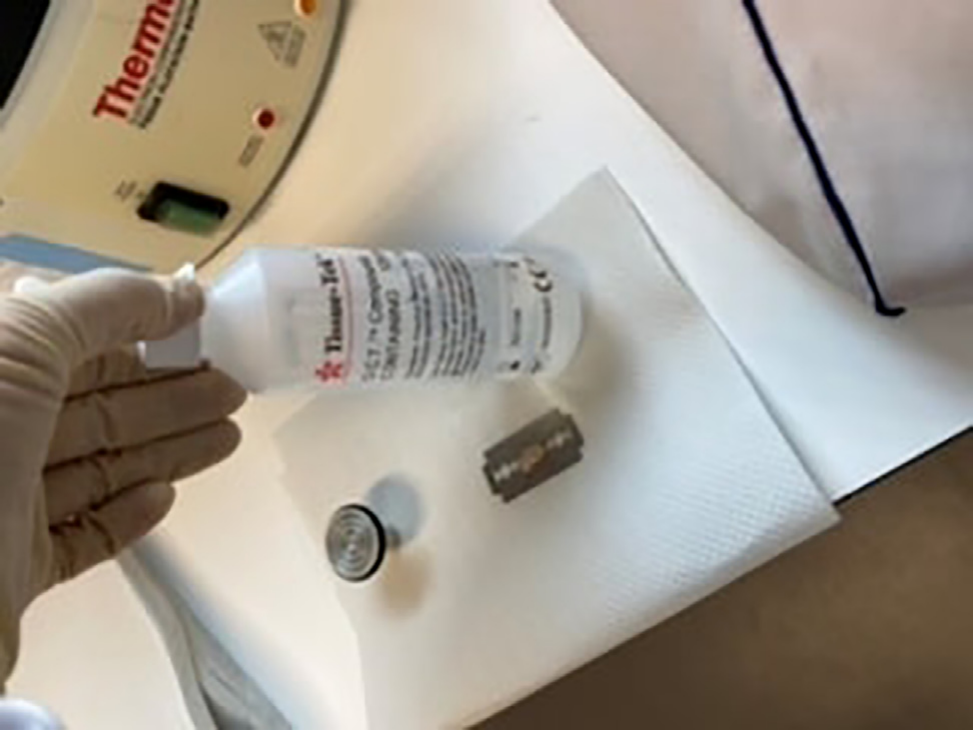
16. Place it in its holder inside the cryostat to make sure it is at – 20°C.
17. Change the blade in the cryostat machine and make sure it is straight.

**Figure:**
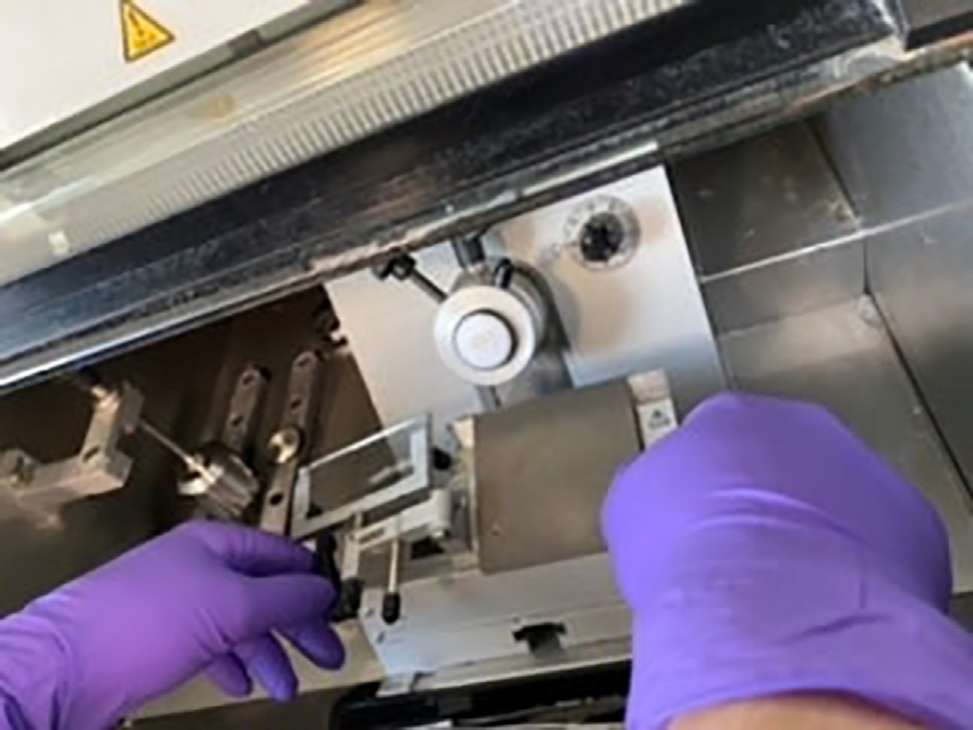
18. Add the holder with the macro-section and lock it when the OCT is frozen.
19. Set the cutting thickness to 10 μm in the cryostat and cut until the whole section is completely exposed.
20. Set the cutting thickness to 50 μm in the cryostat.

***Note:*** You can do some trial sections to make sure the sections are straight, depictable by the appearance of the whole brain section, and smooth, defined by the absence of holes, stripes or scratches. Inspect the cut sections and make sure it is cutting in a straight line.

21. Place the slides for collection of tissue as close to the collection platform as possible and have a brush ready to collect the cut sections. Continue until all of the macro-sections are cut into smaller 50 μm sections.
22. Place the labeled slides vertically in a slide box at – 80°C until further usage.

**Pause Point:** [To ensure that the brain sections are completely frozen, we recommend keeping it at – 80°C for 1 day, but longer storage is also possible until further use.]

### Immunofluorescent staining protocol – Day 1

**Timing:18.5 h**

This step is needed to wash, permeabilize, block and incubate with primary antibody to stain IBA1+ cells.

23. Put the glass slides with the tissue sections on a piece of paper and defrost for 10 min.
24. Add the glass slides to a coplin box immersing the slides in 1x PBS for 5 min two times.
25. For permeabilization and peroxidase removal: Immerse the slides in PBS with 3% H_2_O_2_ + 1.5% Triton-X-100 for 30 min (The Permeabilization and peroxidase removal buffer, see materials and equipment).
26. Wash 1 × 5 min in PBS with 0.1% Triton-X-100 and 1 × 5 min in 1x PBS.
27. Blocking: Immerse slides in 5% BSA in PBS for 30 min.
28. Rinse the slides and remove as much liquid as possible with KimWipes.
  a. Incubate slides horizontally and add 300 μL of 2% BSA in 0.3% Triton-X-100 with primary antibodies (1:1000) for 17 h overnight at room temperature.
  b. Cover the solution with Parafilm cut into sections covering the whole glass slide.
29. Soak some paper tissue with water and place it into the box to make sure that the slides do not dry out.

### Immunofluorescent staining protocol – Day 2

**Timing: 3.5 h**

30. Gently remove the Parafilm with forceps and inspect the sections.
31. Wash 3 × 10 min in 1x PBS 0.3% Triton-X-100 in a coplin box.
32. Quickly dry the slides to remove all liquid surrounding the tissue and if there is still remaining liquid, gently wipe it off with Kim wipes.
33. Add the slides to the staining box horizontally and add 300 μL of secondary antibody (1:1000) solution in 2% BSA in 0.3% Triton-X-100.
34. Incubate the slides at room temperature for 2 h covered from the light.
35. Gently remove the Parafilm and wash 3 × 10 min in 1x PBS.
36. Dry remaining liquid with Kim wipes and add one drop of mounting media containing DAPI before adding the glass cover slide on top.

***Note:*** Use of cotton swabs is helpful to remove liquid surrounding the tissue. Make sure there are no bubbles in the mounting medium. If bubbles, gently push on the glass cover to get the bubbles out from the mounting media.

37. Let the glass slides dry at room temperature for at least 2 h and fix the glass cover to the Epredia slides.
  a. When dry, add nail polish on each corner or along the sides to fix the glass slides to the Epredia slides.
  b. Keep at room temperature for 12–18 h to make sure it is completely dry.

### Imaging – Day 3

**Timing: 2–2.5 h acquisition per section**

38. Inspect your glass slides and clean the slides gently with ethanol, in case of dust or condensation.
39. Start the LSM 880 confocal microscope and place the slide with oil and image with the LD LCI Plan-Apochromat 25 × oil objective.
40. Create one track with laser 405 nm (21.7) and 633 nm (36.1) with a pinhole of 19.8, 0.69 Airy Units 0.6 μm section. DAPI: gain: 840, digital offset: 1, digital gain: 0.6 and Far red A647: gain: 655, digital offset: - 1, digital gain 1.5 respectively.

***Note:*** The microscope settings can be adapted according to the staining. See the note below, limitations and troubleshooting steps. It is important to keep the exact same settings for all images to compare the intensity of the staining. If you are using a different microscope, it is possible that you would need to adapt the settings to enhance visualization of microglia throughout the tissue and to minimize background noise. Make sure that you use the correct filters that matches the emission spectrum of the fluorophore used, as well as the correct excitation light wavelength (see point 40). You can increase the exposure time if the staining is weak in the middle of the 50 μm tissue section. Adjusting the pinhole will also increase the intensity of your staining. Adjust gain to amplify the signal from the stained regions without amplifying background noise, set the offset to reduce background and improve contrast. Just always make sure that the exact same settings are used if you want to compare the intensity across all sections.

41. Change the settings in the black Zen software to acquire three by three tile scans for a total of nine tiled images (resolution 0.221 μm in x and y dimension, 1 μm z dimension) with a 10% overlap.

***Note:*** Slow scan speed can increase the signal-to-noise ratio for dim stained tissue. Be aware that nine tiles of 28 stacks will take around 2 hours for acquisition of each image with a resolution of 0.221 μm in x and y dimension and 1 μm z dimension. It is possible to increase the scan speed and lowering the resolution, but we would not recommend to go below 28 stacks as a microglia cell ranges from 5-15 μm in diameter and processes can extend over a larger volume and area. It is also possible to reduce the number of tiles to get shorter acquisition times, but note that the number of microglial cells will then decrease. Averaging multiple scans can help reduce the noise in fluorescence staining.

42. Open the Z-stack tool in the black Zen software and check the quality of the staining in the z-stack and pick around 28–32 stacks and aim to have a range of 30 μm. Each image will have a size of 1020.23 μm containing 4608 pixels horizontally and vertically.

***Note:*** An even better resolution will be obtained by doing step-sizes of 0.5 μm, however this will increase the acquisition time. In this protocol, an average thickness per 3D image was 30 μm (30 stacks of 1 μm steps), as this is the size of a microglia, so this is what we recommend. We strongly discourage working with a lower resolution (e.g. 10x). We tested these parameters at 40x and they work well – you typically have way more robustness when the resolution is much higher.

The only parameter that needs to be changed when working on 40x is the 3d voxel size filtering. See comment on step 46d.

43. After acquisition, store slices at − 80°C. The slides should ideally be acquired within a maximum of 3 weeks after the staining but can, in our experience, be acquired up to 6 years later.

### Applying microglia and immune cell morphological analysis and clustering to images

**Timing: 2 h per acquisition**

In this section, the image-processing pipeline is fully described. This will allow the user to generate a dataset that will contain morphological information about microglial cells present in the raw microscopy acquisitions.

44. Open each individual acquisition in Imaris and inspect each image by using both the Surpass and Slice View mode.

***Note:*** Consider removing from the analysis acquisitions where the following problems have occurred: holes/ruptures in the tissue, tiles misalignment, dim signal in more than four stacks and strongly uneven cell distribution throughout the 3D acquisition. See troubleshooting step one to five.

45. Correct the slice intensities with the *Normalizer Layer* function, followed by a *Gaussian Filter* (width = 0.3), both available under the *Image Processing* section.

**Figure:**
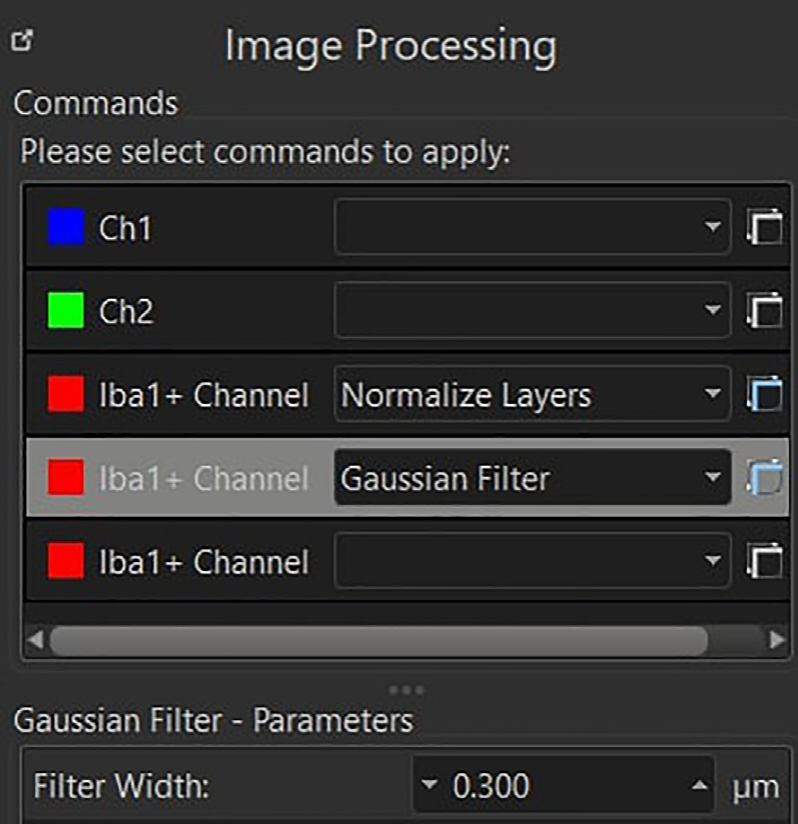
46. Perform the segmentation by using the *Surface Creation* Tool.

**Figure:**
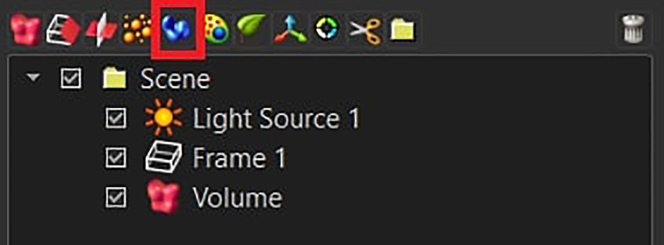

  a. Deselect *Classify Surfaces* and deselect *Object to Object Statistics* and press next.

**Figure:**
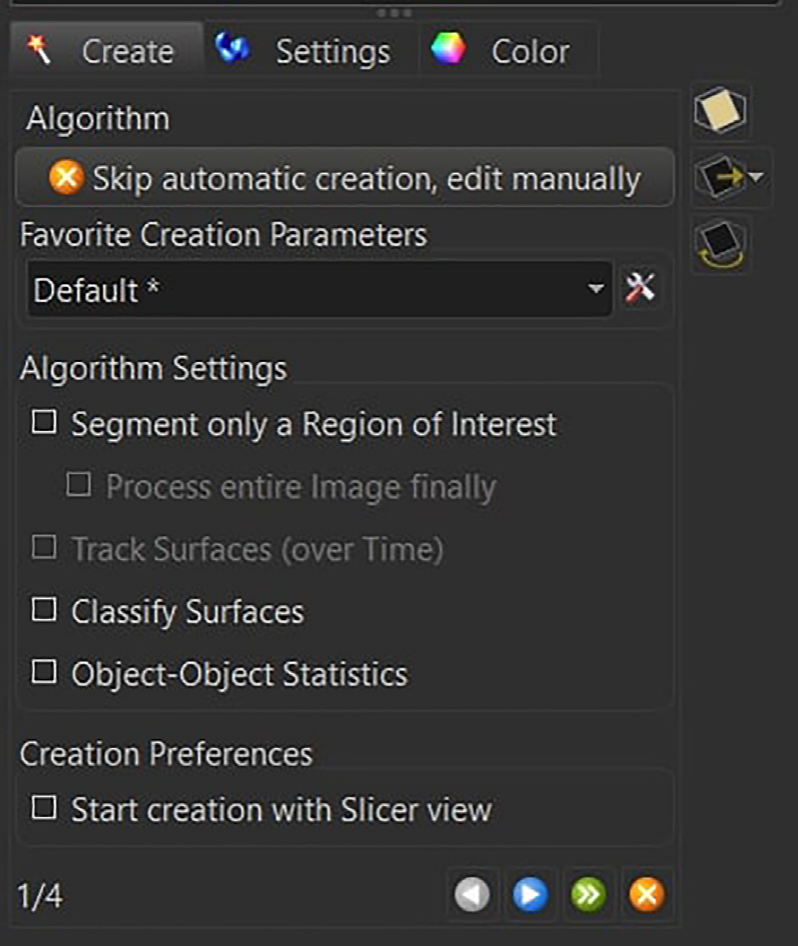
  b. Select the correct Iba1 channel and enable the *Local Contrast* with a diameter of 0.5 μm, as well as the *Smooth* option with a *Surface Detail* parameter of 0.5 μm.

**Figure:**
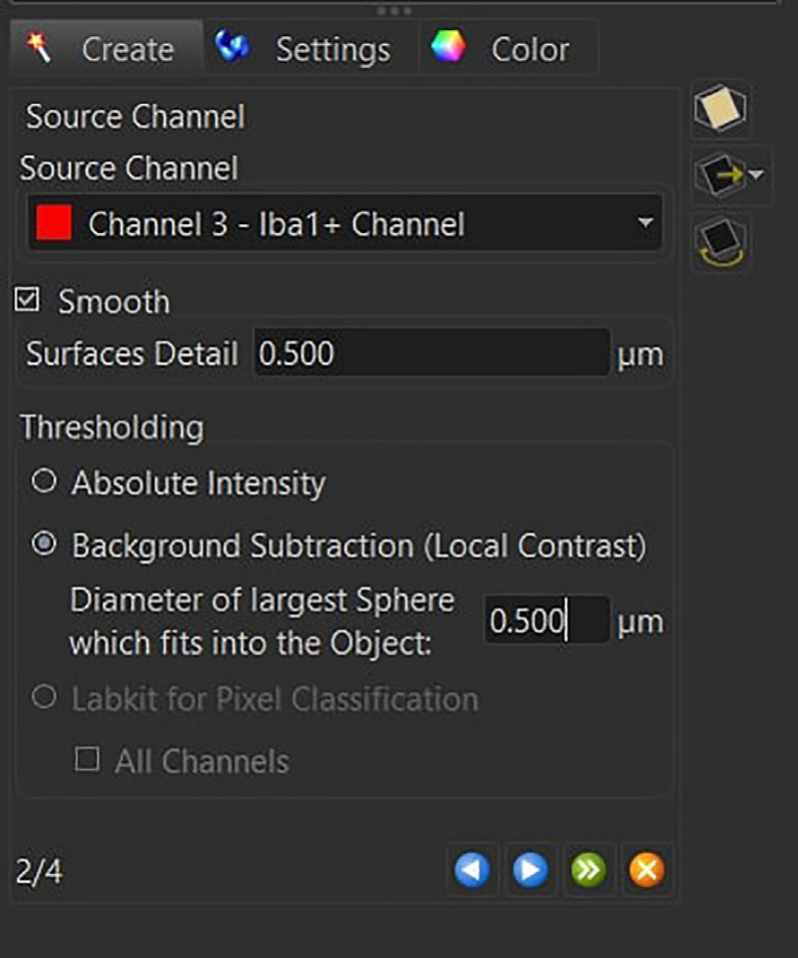

Press next.
  c. Set the background subtraction threshold between 0.5 and 1.5.

**Figure:**
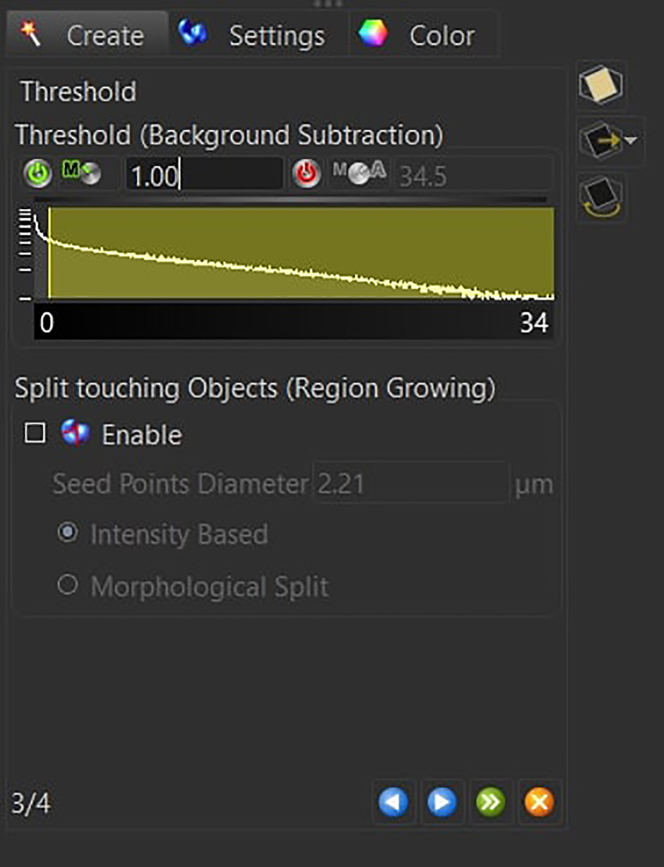

Keep the *Split Touching Object* option disabled and press next.
    ***Note:*** The background subtraction threshold is manually adjusted to account for image-to-image variation (between 0.5 and 1.5). To set this parameter, check that the gray surface is wide enough to have the microglia branches connected to the soma, while at the same time not overestimating the microglia size with respect to the signal coming from the staining.
  d. Filter the resulting 3D structures by voxel size (usually between 18′000 and 180′000 for mouse microglia at 20x).

**Figure:**
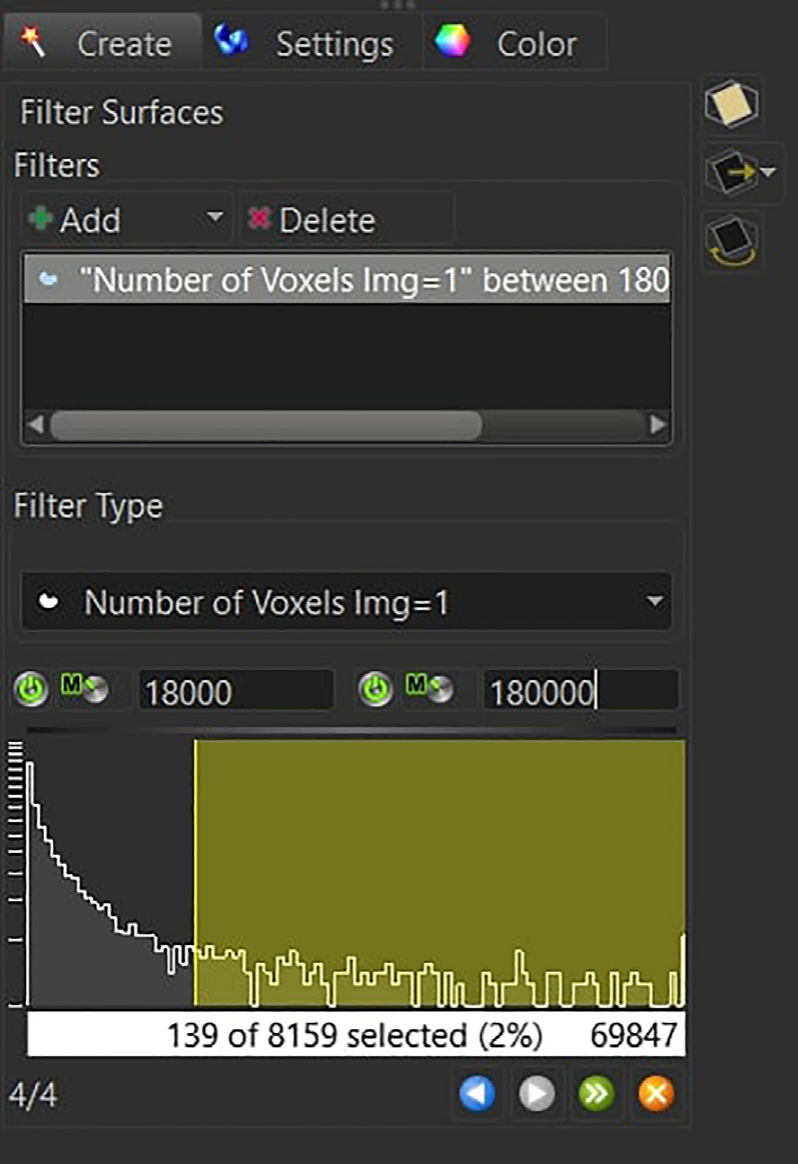

Press the finish button.
    ***Note:*** The user can visualize in real time which structures will be kept and which structures will be removed. By dynamically changing the upper and lower voxel size threshold, pay attention to the removal of isolated branches, cells that have been partially cut off by the first or last slice of the acquisition, and aggregations of microglia that are not identified as individual cells.
47. Select the newly created surface on the top left menu and open the *Edit* section

**Figure:**
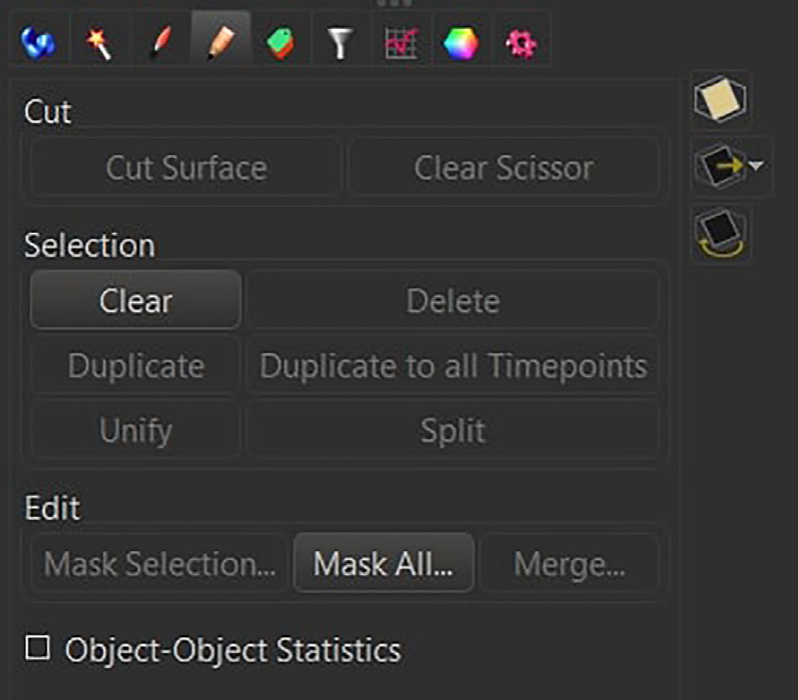

and mask the segmentation to the corresponding Iba1 channel by setting the voxel intensity outside the surfaces to 0 and inside the surfaces to 255.

**Figure:**
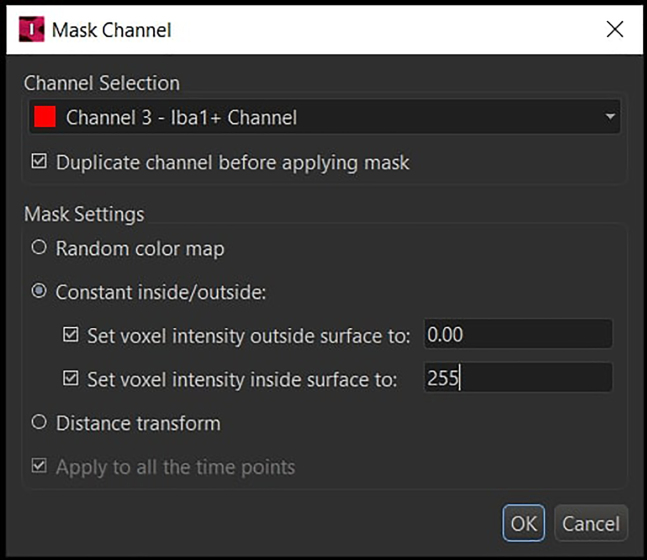
48. Remove all the other channels except for the newly created masked channel (under Edit -> Delete Channels).

**Figure:**
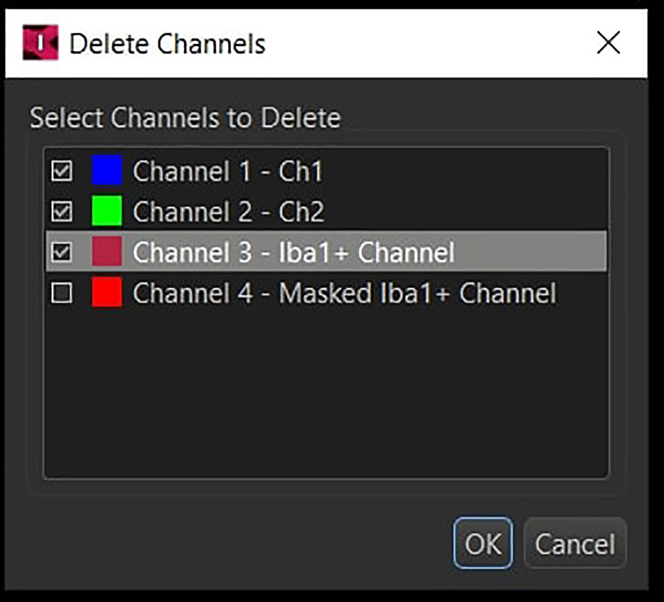

Finally, export the image in *.ims* format.

**Figure:**
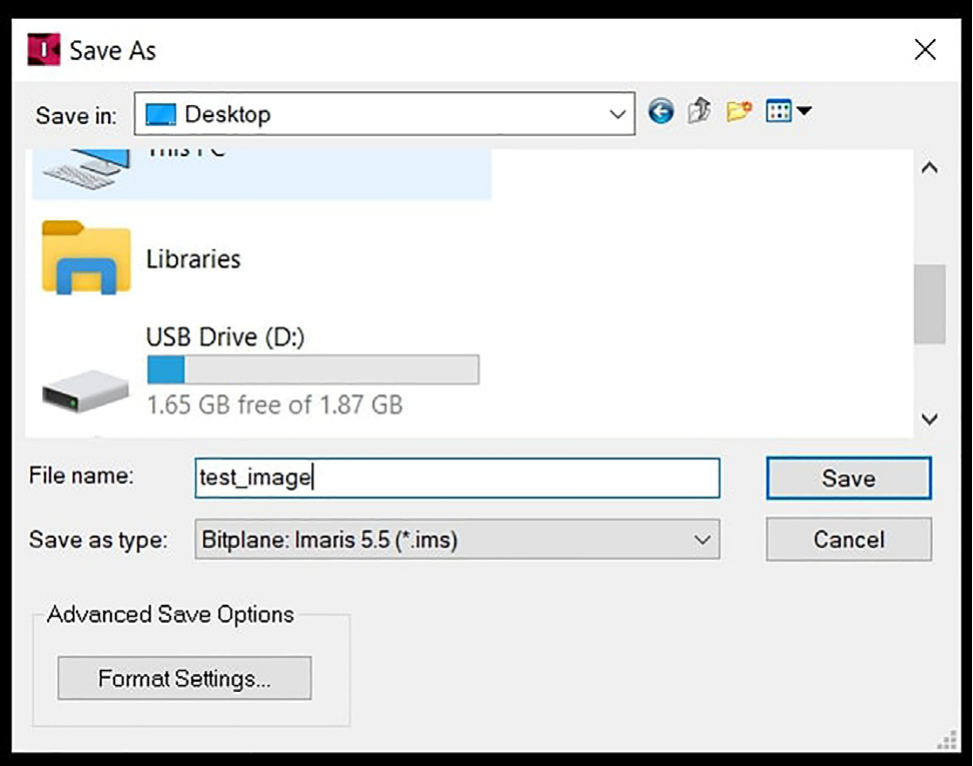
49. Open MATLAB and load the *FeatureExtract.m* script (https://github.com/CorradoAmeliLU/MIC-MAC-2.1

**Figure:**
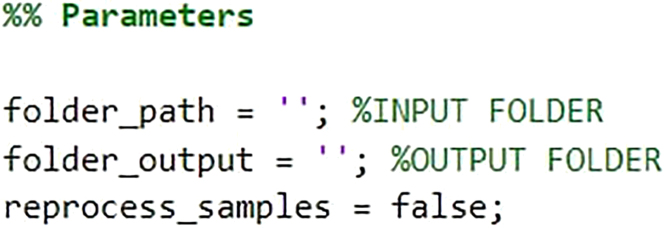

and run the script.

***Note:*** This script will take as input one or more Imaris images and quantify several morphological features for each cell. Set the parameters at the beginning of the script based on the folder that contains the segmented Imaris file/s, the output folder, and whether you want to reprocess files that have already been processed (TRUE or FALSE option).

The user will be able to track the progress of the procedure by looking at the messages appearing on the MATLAB console. For each acquisition, two MATLAB data files will be created. The *_attributes.mat* file contains a table where each row contains information about one single cell and each column represents a morphological feature. The *_volumes.mat* contains a logical 3D matrix for each row that encodes each individual microglial cell full morphology. Note that the row ordering between the two files is maintained, meaning that the n^th^ microglia on the attribute table will correspond to the n^th^ microglia on the table containing the microglia morphologies e.g., the microglia in the 10^th^ row in the attributes.m-at table will correspond to the same microglia in the 10^th^ row in volumes.mat. A tutorial is available on the GitHub repository.

50. Load the newly generated Matlab data on any analysis tool the user is familiar with and proceed to analyze the data.

## Expected outcomes

This protocol is useful for evaluating 3D microglial cell morphology and density in mouse brain sections. In total, 16 morphological features of microglia (1^st^ largest bound, 2^nd^ largest bound, 1 over 2, average curvature, average node degree, compactness, ending node density, link density, max curvature, max edge length, mean edge length, node density, polarity, S-metric, volume, volume/number of edges) can be extracted, analyzed, and compared between different mouse models, brain regions, treatments and between sexes and age groups.

You can expect information from 300-350 cells per 9 tile scan when using this protocol. Additionally, various versions of this protocol can be adapted to accommodate both *in vivo* morphology and transcriptional analyses from the same mouse.

Examples of microglia morphologies with different values for each morphological feature, as well as an example of a comparison between different conditions can be found in the previous study.^2^

## Quantification and statistical analysis

The analytical approach must align with the structure of the experimental design, ensuring that each level of the data hierarchy is properly accounted. In cases where treatments are applied at the animal level, the animal serves as the experimental unit, and the variability between animals provides the basis for hypothesis testing (parametric or non-parametric, depending on normality and homoscedasticity assumptions). When analyzing cell-level features, averaging across multiple cells within an animal helps to mitigate intra-sample variability, but it is crucial to recognize that these cells do not represent independent replicates. For studies focused on classification or subtyping of cells, algorithms such as k-means can be employed to identify distinct cell types or morphological subgroups, and indeed this family of analyses would be performed at individual cell level. Lastly, if a study inquires how multiple factors are influencing the outcome, regression models can be applied, incorporating these factors as independent variables to quantify their specific contributions.^5^ This approach tailored to the study design ensures that statistical analyses reflect the real structure of the data, avoiding inflated significance and providing more accurate, interpretable results.

To summarize, the adoption of the proper statistical analysis to inspect the dataset strictly depends on the hypotheses that the user is willing to test and the associated assumptions. Two examples are presented in previous studies.^1^^,^^2^

## Limitations

In case of mouse models with brain infiltrating monocytes, this method will not distinguish between resident microglia and monocyte-derived macrophages. To delineate microglia morphology in models with expected immune infiltration, the addition of the antibody against TMEM119 to distinguish microglia from macrophages may be envisaged.

For characterizing mouse models with large plaques, such as in Alzheimer’s disease models, this protocol will automatically exclude large areas of clumps of cells via the voxel filtering. Therefore, to quantify microglial cell morphology in brain tissue containing large plaques, the majority of cells within the plaques will be excluded.

It is likely that slight changes in microscope laser settings will be needed to acquire a bright image throughout the stacks of a total of 30 μm and therefore we would not recommend to use the intensity parameter to analyze microglial activation, unless the exact same settings were used to compare all the images.

In addition, the expected outcome may vary based on the default settings of the microscope and between microscope brands. Make sure to image all slides on the same microscope with the same settings and be aware of inter-laboratory variations due to different microscopes and default laser settings.

## Troubleshooting

### Problem 1: Holes visible in tissue

As shown in the image below, holes in the tissue should not be seen in acquired images of tissue sections, as information will be lost. This can either be a problem of over- or under-fixation.

**Figure:**
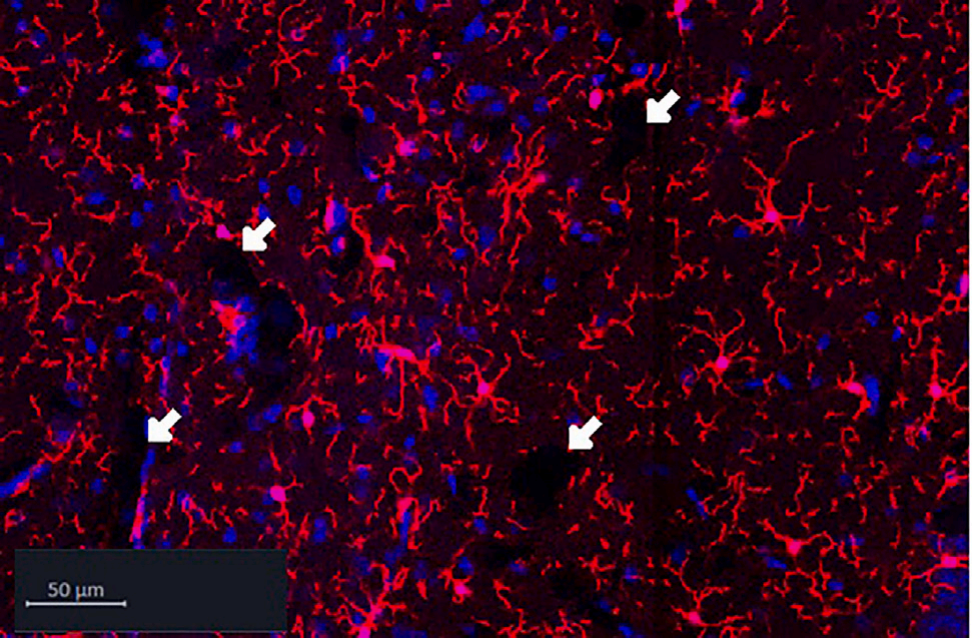

### Potential solution

Increase or decrease the 4% PFA immersion fixation time of the tissue. See step 7 “Fixation and embedding”.

Immersion in 4% PFA at 4°C usually requires 24–48 h and is dependent on the volume of the tissue. PFA has a penetration rate of approximately 1 mm per hour at room temperature, however fixation is slower at 4°C, but it is necessary to keep the brain cold to preserve antigenicity for immunohistochemistry. Given that a mouse brain is approximately 1 cm^3^, fixation of one hemisphere will take around 24 h and between 24-48 h for a whole mouse brain.^6^

Holes in tissue could also be caused by a too strong permeabilization. See step 25 in immunofluorescent staining protocol day 1. Decrease the percentage of Triton-X-100 to e.g., 1% or 0.5%. It can also be necessary to decrease the percentage of Triton-X-100 concentration for tissue thinner than 50 μm. Alternatively, use 1.5% Tween 20, which is a more gentle detergent than Triton-X-100.

A third potential solution could be to perfuse with cold 4% PFA as this will ensure rapid and thorough fixation. However, this step would require an animal protocol giving permission to perfuse animals with fixatives.

### Problem 2: Striped lines visible in tissue sections

**Figure:**
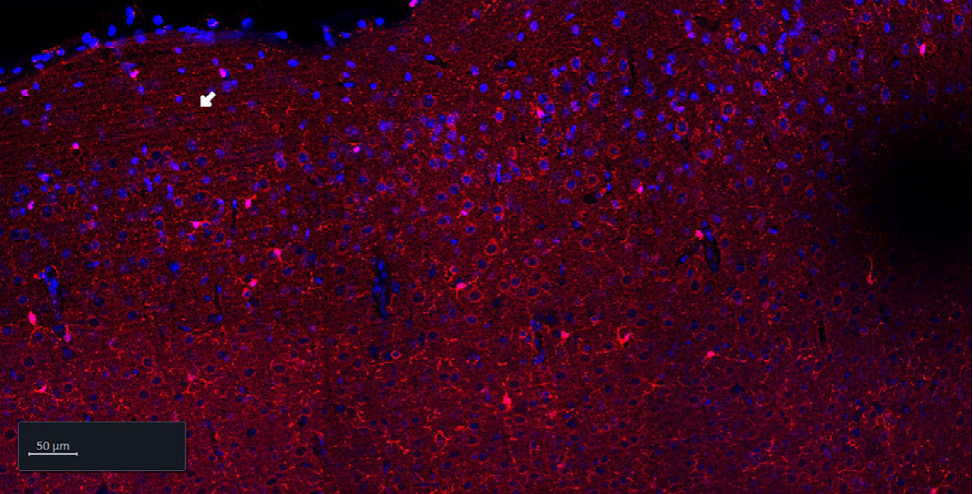

### Potential solution

If many striped lines are visible, it is usually due to a suboptimal tissue cutting on the cryostat. You can solve this by frequently changing the blade for cutting, a better placement of the tissue with OCT before cutting and by always keeping the slides, the blade and the tissue at − 20°C. The cutting can become uneven or leave striped marks if the tissue is not cold enough. Take some breaks and make sure that the cryostat cools down before continuing cutting. Furthermore, make sure that the cut is done in one smooth movement and to not stop the cryostat handle while cutting. This protocol requires a cryostat, but other methods using a vibratome and free floating sections as previously described^7^ can be used as well prior to applying microglia and immune cell morphological analysis and clustering to conduct microglial morphological analyses.

### Problem 3: Segmentation of cells not aligned between tiles/Tiles misalignment

As illustrated in the image below, microglial cell processes are cut from one tile to the other. This is a mechanical issue when moving the slide under the laser during image acquisition or it is an issue of processing of the image in the Zen software.

**Figure:**
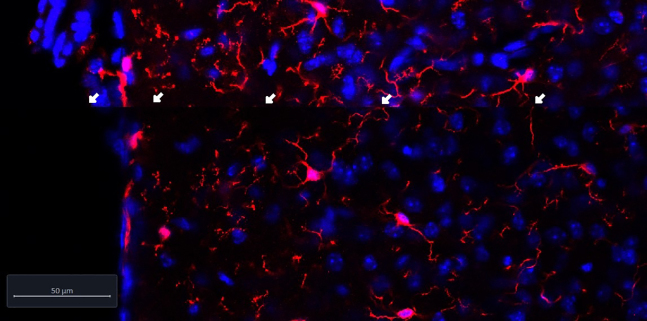

Methods video S1

Methods video S1. Tile misalignment leading to incomplete microglial cells Problem 3, Tile Misalignment, Troubleshooting 3, related to troubleshooting step 3.

### Potential solution

Make sure to always acquire the tissue section using tile scans with a 10% overlap between tiles to avoid this issue.

Follow the Zeiss guide, Chapter 1 – SYSTEM OPERATION paragraph 5.2.19 Multidimensional Acquisition – Tile Scan. 5.2.20 Scan Overview Image for Tiling.

### Problem 4: Unspecific binding of antibodies to tissue tear

**Figure:**
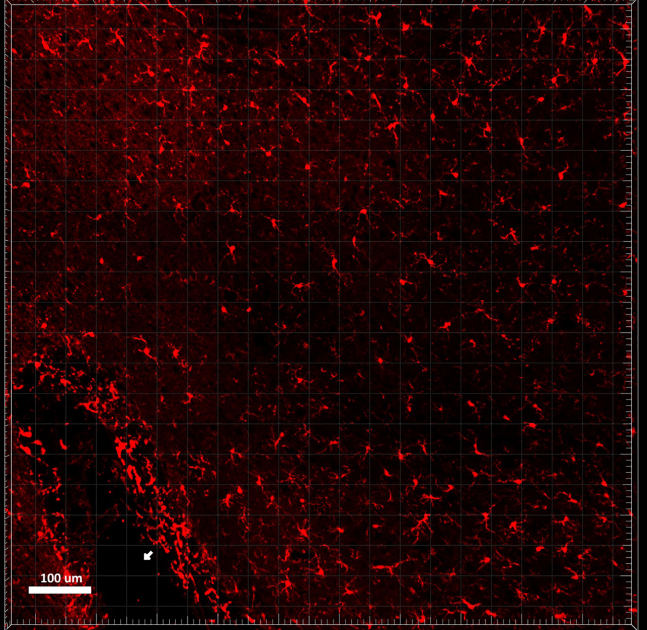

### Potential solution

If you still want to include the image because you cannot avoid the tear of the specific tissue section of interest, you can manually remove unspecific binding in Imaris. Zoom in and check each recognized microglia cell in the image. Remove what is clearly unspecific binding and save the image for further processing. However, we would recommend avoiding including images of tissues with unspecific binding at a border or a tear.

### Problem 5: No or extremely dim signal in tiles in more than 4 stacks

**Figure:**
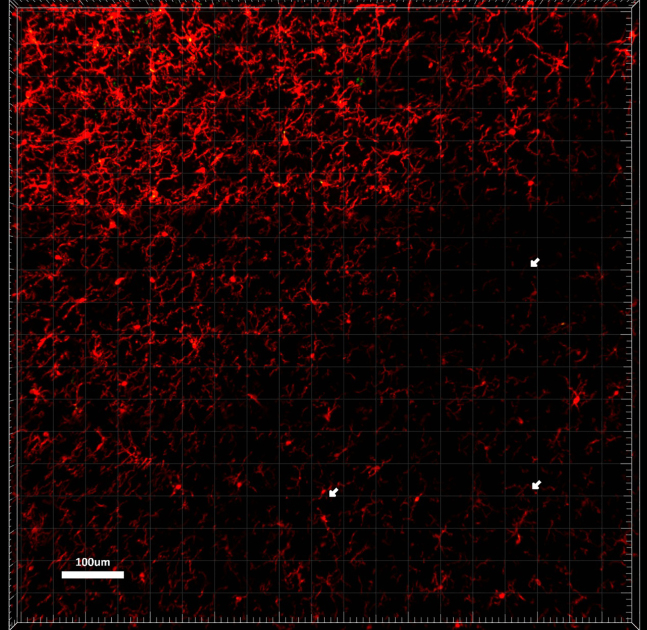

### Potential solution

As 30 μm tissue stacks are challenging, it is not unusual to encounter uneven antibody penetration throughout the tissue section. If tiles of more than four z-stacks are very dim or completely dark, the image should be excluded as the information of many cells will be lost. A potential solution could be to increase the time of permeabilization or the percentage of Triton-X-100 to 2%. We do not recommend using a higher concentration of antibodies.

## Resource availability

### Lead contact

Further information and requests for resources and reagents should be directed to and will be fulfilled by the lead contact, Alessandro Michelucci, Alessandro.michelucci@lih.lu.

### Technical contact

Technical questions on executing this protocol should be directed to and will be answered by the technical contacts, Frida Lind-Holm Mogensen, Frida.lind-holm@lih.lu, or Corrado Ameli, Corrado.ameli@gmail.com.

### Materials availability

This study did not generate new unique reagents.

### Data and code availability

Datasets are available in Lind-Holm Mogensen et al.,^1^ Fixemer et al.,^2^ and Salamanca et al.^3^ The code, including a guide, is available via GitHub: https://github.com/CorradoAmeliLU/MIC-MAC-2.1. An illustrative acquisition, as well as the intermediate and final results of the computational part of the protocol, are available via OwnCloud: https://owncloud.lcsb.uni.lu/s/qoXE9SmgIZ34xvX/authenticate. The credentials needed to download the files from OwnCloud are available in the aforementioned GitHub repository.

## Acknowledgments

The Luxembourg National Research Fund (FNR) supported F.L.-H.M. and C.A. through the FNR-PRIDE programs i2TRON (PRIDE/14254520/I2TRON) and CriTiCS (PRIDE/10907093/CRITICS), respectively. Additionally, C.A. was supported by the Caisse Médico-Complémentaire Mutualiste Luxembourg (CMCM) matching grant. For the purpose of open access, and in fulfilment of the obligations arising from the FNR grant agreement, the author has applied a Creative Commons Attribution 4.0 International (CC BY 4.0) license to any Author Accepted Manuscript version arising from this submission. We would like to acknowledge Ms. Virginie Baus and Mr. Andrea Scafidi for assistance with the cryostat, Dr. Djalil Coowar at the University of Luxembourg Animal Facility for mouse breeding and handling, Ms. Céline Hoffmann for assistance with the confocal microscopy, and Ms. Amandine Bernard for helping with the key resources table. Lastly, we would like to acknowledge Dr. Eduardo Rosales Jubal for assistance with statistical analyses. The figures and the graphical abstract were created using BioRender.com.

## Author contributions

F.L.-H.M. optimized and carried out all animal work, tissue preparation, staining, and microscopy acquisitions. C.A. optimized and carried out segmentation and computational analyses. A.S. and A.M. conceptualized and supervised the project. F.L.-H.M. and C.A. wrote the manuscript draft. All authors reviewed and edited the manuscript.

## Declaration of interests

The authors declare no competing interests.
